# TRFs and tiRNAs sequence in acute rejection for vascularized composite allotransplantation

**DOI:** 10.1038/s41597-022-01577-y

**Published:** 2022-09-07

**Authors:** Yuan Fang, Haibo Li, Jingting Chen, Yao Xiong, Xu Li, Binbin Sun, Shengli Li, Jianda Zhou, Shoubao Wang

**Affiliations:** 1 Department of Plastic Surgery, The Third Xiangya Hospital, Central South University, Changsha, Hunan China; 2 Department of Plastic and Reconstructive Surgery, Shanghai Ninth People’s Hospital, Shanghai Jiao Tong University School of Medicine, Shanghai, China; 3 College of Chemistry, Chemical Engineering and Biotechnology, Donghua University, Shanghai, China

**Keywords:** High-throughput screening, Predictive markers

## Abstract

Illumina tRFs & tiRNAs-seq analysis was used to characterize the whole transcriptomes of acute rejection caused by vascularized composite allotransplantation (VCA). tRFs & tiRNAs-seq information for muscle samples with VCA was obtained and compared with similar information for same age- and sex-matched healthy control subjects. The expression of 16 tRFs and tiRNAs, including 5 up-regulated target genes and 11 down-regulated target genes, were significantly different. According to bioinformatics analysis and reverse transcription quantitative polymerase chain reaction, we speculate that tiRNA-1-34-Glu-CTC-1 plays an important role in VCA-induced acute rejection by regulating the *CACNA1D* gene in the MAPK signaling pathway The findings provide the whole-transcriptome signatures of acute rejection for VCA, allowing further exploration of gene expression patterns/signatures associated with the various clinical symptoms of acute rejection for VCA.

## Abstract (summary)

**Table:** 

Measurement(s)	gene expression
Technology Type(s)	RNA sequence
Factor Type(s)	RNA
Sample Characteristic - Organism	rn6
Sample Characteristic - Location	China

## Background & Summary

The development of microsurgery has greatly advanced vascularized composite allotransplantation (VCA). VCA is used to repair large-area defects that cannot be achieved by traditional reconstruction techniques. Some believe that VCA technology remains experimental, but some cases of very successful hand and face transplantation using VCA surgery have been reported^1,2^. To prevent rejection after the operation, recipients usually receive routine immunosuppressive therapy. However, some patients will experience acute rejection attacks even with immunosuppressive therapy^3^. VCA, like organ transplantation, faces problems such as long-term survival and dysfunction. Thus, in such cases, it is very important to explore the molecular mechanisms of acute rejection in VCA. This data will help in early detection and rescue so as to reduce complications and improve the success rate.

Recent studies have reported that tRNAs are excised from different sites to produce different fragments. These small fragments form a heterogeneous population of small noncoding RNAs (ncRNAs) called tRNA-derived small RNAs (tsRNAs). These small fragments have been previously considered to be tRNA degradation products. However, a growing number of studies have attributed multiple functions to this new ncRNA. Based on their lengths and cleavage sites, tsRNAs can be roughly divided into two types with differing functions: tRNA derived stress-induced RNAs (tiRNAs) and tRNA-derived fragments (tRFs). RNase angiopoietin cleaves mature tRNA to produce 31–40 nt long RNA strands, which are known as tiRNA. tiRNA can accumulate after oxidative stress, heat shock, ultraviolet radiation, or hypoxia^4^. tRFs are between 14–30 nt and can be further subdivided into tRF-5, tRF-3, and tRF-1 according to their excision positions. tRFs are related to the regulation of gene expression and protein translation^5^.

**Fig. 1:**
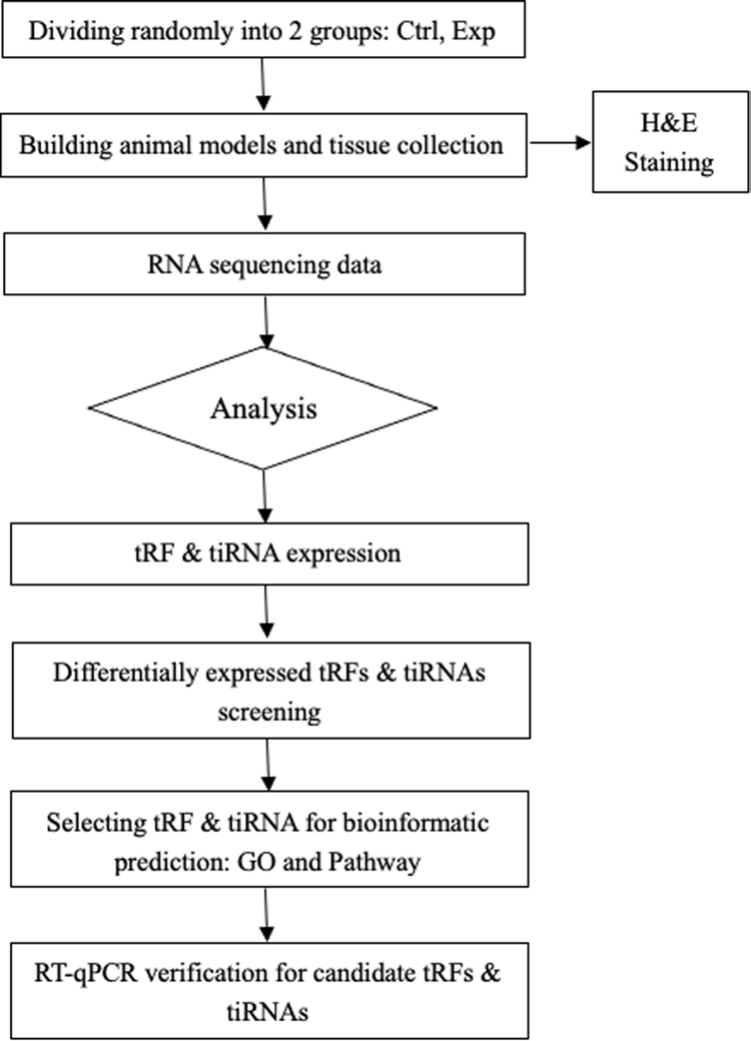
tRF & tiRNA-seq experiment workflow.

Currently, the role of tRFs and tiRNAs in various diseases has attracted the attention of many researchers. However, the relationship between tRFs and tiRNAs and acute rejection in VCA has not received much attention. Therefore, the purpose of this study is to explore the expression profiles of tRFs and tiRNAs and to determine their molecular mechanisms in acute rejection of VCA. The flow chart of this study is given in Fig. 1.

## Methods

### Animals model

The VCA model was constructed via the rodent hindlimb allograft model. For this study, Shanghai Sippr-BK Laboratory Animal Co. Ltd. (Shanghai, China) provided male Lewis and male Brown Norwegian rats weighing 200–250 g. These different rats received one hindlimb from the same donor. All animal procedures were approved by the ethics committee of the Ninth People’s Hospital Affiliated to Shanghai Jiao Tong University School of Medicine. This work was carried out in strict accordance with the guidelines of the Laboratory Animal Manual of the National Institute of Health Guideline to the Care and Use of Animals.

**Fig. 2:**
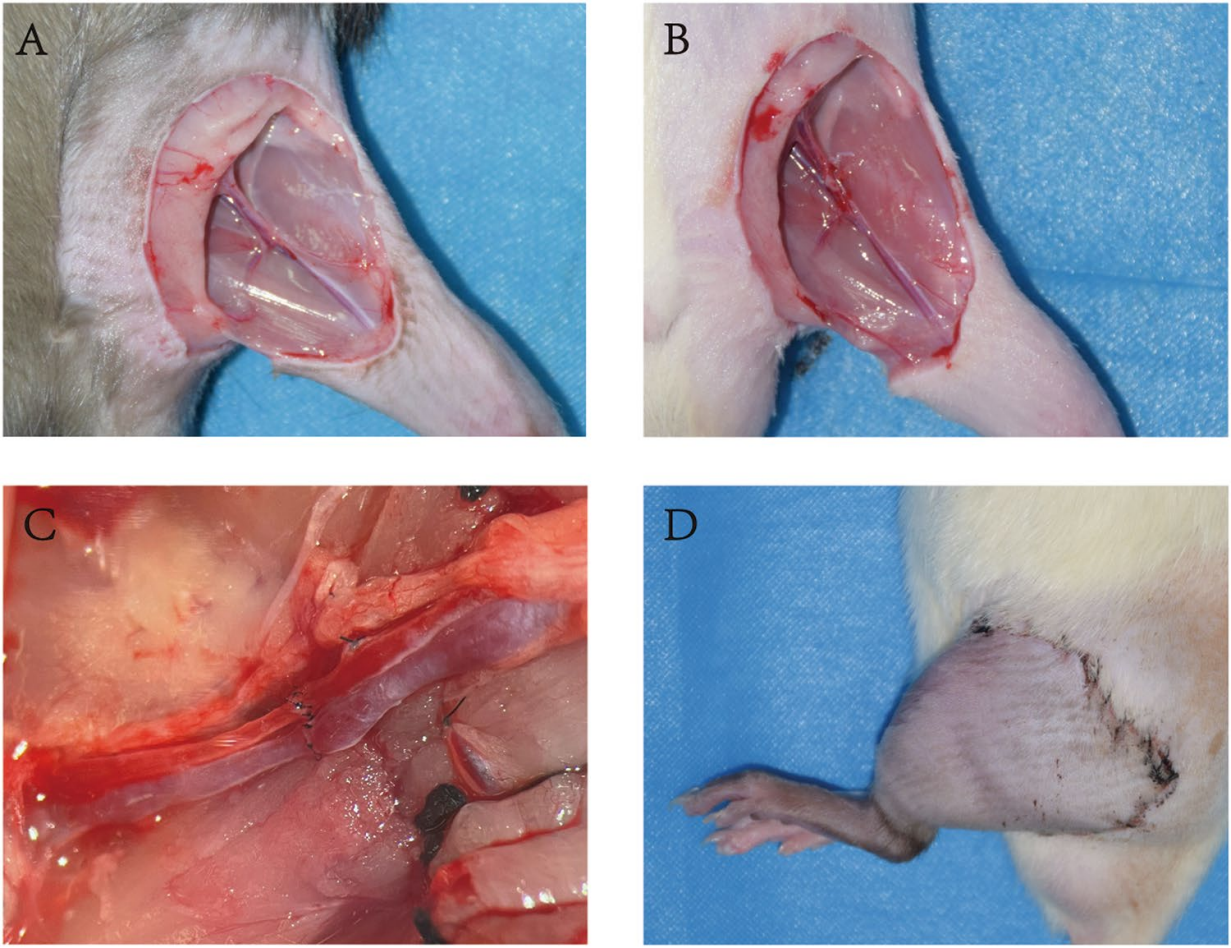
Surgical procedures. (**A**) Prepare the donor site for amputation. (**B**) Prepare the receptor. (**C**) Perform vascular anastomoses. (**D**) Close muscles and skin.

Before surgery, the rats were deeply anesthetized using continuous inhalation of isoflurane. The operation was performed as described previously^6^. In short, the two hind limbs of the donor rats were amputated in the middle of the thigh. Before amputation, the limbs were perfused with cold heparinized Ringer’s lactate solution and then stored in cold heparinized Ringer’s lactate solution until transplantation. Each collected hind limb was transplanted *in situ* to isolated recipient rats. The femur was fixed with an 18 G intramedullary needle. The dorsal muscles and skin were sutured with 3-0 silk thread. Afterwards, under a microscope, the femoral artery, vein, and sciatic nerve were anastomosed with 12-0 nylon thread. After confirming that the anastomotic vessels were clear, the ventral muscles and skin were sutured with 3-0 silk thread (Fig. 2). We did not evaluate motor and sensory functions in this study.

### Tissue collection and preservation

Male BN rats were used as allograft donors. Donors were transplanted into male Lewis rats. The preoperative muscle tissue of the rats was used as the control group, and the postoperative muscle obtained tissue on the 7th, 10th and 14th day was used as the experimental group. The tissues were used to analyze the preliminary mechanisms of acute immune rejection.

Removed skin tissue specimens were divided into two parts and stored. Half of the skin specimen was cut into pieces and frozen in liquid nitrogen for future use. The other half of the tissue specimen was placed in an EP tube and stored on dry ice for molecular detection. Each skin sample was marked with a corresponding label.

### RNA extraction

Add 1 ml of TRI reagent to every 50–100 mg of tissue sample and homogenize with an electric homogenizer. After homogenization, the samples were incubated at 15 to 30 °C for 5 minutes. Add 0.2 ml of chloroform to every 1 ml of TRI reagent homogenate sample, manually shake the tube vigorously for 15 seconds, and incubate at 15 to 30 °C for 2 to 3 minutes. 12000 × g at 4 °C centrifuge for 15 minutes. The aqueous phase was mixed with isopropanol to precipitate RNA. After mixing, it was incubated at 15 to 30 °C for 10 minutes, and then incubated at 4 °C for 12000 × g centrifuge for 10 minutes. Remove the supernatant, add 75% ethanol, and clean the RNA precipitation. After oscillation, 4 °C 7500 × g centrifuge for 5 minutes. Remove the ethanol solution and dry the RNA precipitation in air for 5–10 minutes. Add RNase free water to dissolve RNA and incubate at 55 to 60 °C for 10 minutes. The obtained RNA solution was stored at −70 °C.

### Histological assessment

The muscle tissue at the transplantation site was obtained on the 14th day after transplantation. These samples were fixed with 4% paraformaldehyde in 0.1 M phosphate buffer (PB, pH 7.4) for 24 hours. The tissue was embedded in paraffin. Prepare slices of samples (5 μm). The nucleus was stained with hematoxylin and the cytoplasm was stained with eosin. After wiping with a microscope, the images were collected and analyzed. Full muscle samples were evaluated histologically according to a grading system for muscle rejection described by Büttemeyer^7^.

**Fig. 3:**
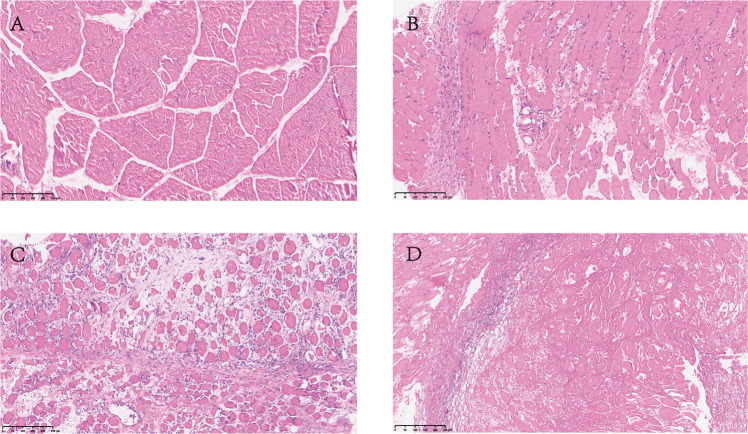
Hematoxylin and eosin staining of muscle samples. Allograft skin tissues at Ctrl (days 0) (**A**) day 7 (**B**) day 10 (**C**) and day 14 (**D**) Bar; 250 μm.

In the allograft group, muscle fiber swelling and patchy focal mild mononuclear interstitial infiltration could be observed on the seventh day post-transplantation. We classified it as Grade I. On the tenth day post-operation, the number of monocytes increased, and some muscle cells were necrotic. We classified it as Grade II. Finally, diffuse lymphocyte infiltration, massive muscle fiber necrosis, and interstitial edema were observed on day 14, which was clearly rejection Grade III (Fig. 3).

### Library preparation procedures

The library was prepared using the NEBNext® Multiplex Small RNA Library Prep Set for Illumina® and the NanoDrop ND-1000 Agilent 2100 Bioanalyzer.

**Fig. 4:**
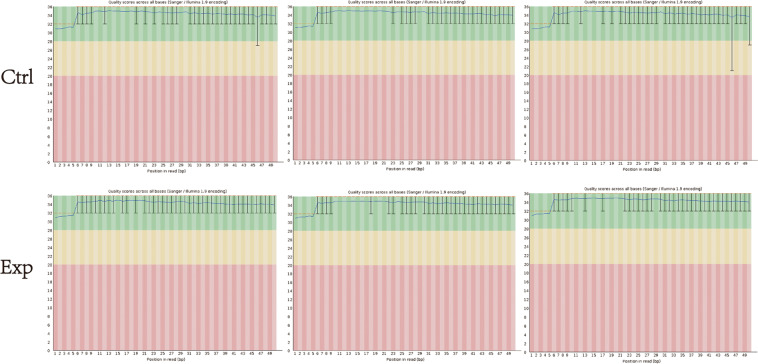
tRF & tiRNA-seq quality score.

Agarose electrophoresis was used to check the integrity of the RNA, which was then quantified on the NanoDrop ND-1000 instrument. RNA sample QC was provided in Fig. 4. To remove RNA modifications that interfere with small RNA-seq library construction, the total RNA samples underwent 3′-aminoacyl (charged) deacylation to 3′-OH for 3′-adaptor ligation. Combine the reagents per deacylation reaction. Mix by vortexing and incubate at 37 °C for 40 minutes. Add 19 μL Deacylation Stop Buffer, mix by vortexing. Incubate at room temperature for 5 min. Then, 3′-cP (2′, 3′-cyclic phosphate) removal to 3′-OH for 3′-adaptor ligation, 5′-OH (hydroxyl group) phosphorylation to 5′-P for 5′-adaptor ligation. Add the reaction components on ice. Incubate at 37 °C for 40 min. Inactivate the Terminal Enzyme by incubating at 70 °C for 5 min. Purify the RNA by phenol-chloroform extraction and ethanol precipitation. Finally, m1A and m3C demethylation for efficient reverse transcription. Remove Demethylase from the freezer, mix by flicking the tube (do not vortex). Briefly spin down the content and place on ice. Set up demethylation mix. Incubate the mix at 37 °C for 2 h. Add 40 μL Nuclease-free Water and then 10 μL Demethylation Stop Buffer (5×) to terminate the reaction. Purify the RNA by phenol-chloroform extraction and ethanol precipitation.

The pretreated total RNA of each sample was used for tRF and tiRNA-seq library preparation. The library preparation procedures included: 3′-adapter ligation, 5′-adapter ligation, cDNA synthesis, polymerase chain reaction (PCR) amplification, size selection of ∼134–160 bp PCR amplified fragments (corresponding to ∼14–40nt small RNAs). The completed libraries were quantified using the Agilent 2100 Bioanalyzer. Based on the quantification results, the libraries were mixed in equal amounts and used for sequencing.

### Sequencing procedures

tRNA sequences of cytoplasmic were downloaded from GtRNAdb^8,9^. tRNA sequences of mitochondrial were predicted with tRNAscan-SE software^10,11^. The DNA fragments from the well-mixed libraries were denatured with 0.1 M NaOH to generate single-stranded DNA molecules and loaded onto the reagent cartridge at a 1.8 pM concentration. The sequencing run was performed on the Illumina NextSeq. 500 system using the NextSeq. 500/550 V2 kit (#FC-404-2005, Illumina) according to the manufacturer’s instructions. The sequencing was carried out by running 50 cycles.

### Data analysis

The image analysis and base calling were performed using the Solexa pipeline (Off-Line Base Caller software, v1.8). The sequencing quality was examined via FastQC, and trimmed reads (pass Illumina quality filter, trimmed 5′, 3′-adaptor bases by cutadapt) were aligned, allowing for 1 mismatch only, to mature tRNA sequences. Reads that did not map were aligned, allowing for 1 mismatch only, to precursor tRNA sequences using bowtie software^12^. The expression profiles of the tRFs and tiRNAs could be calculated based on counts of reads mapped. The sequencing depth (total read counts) for each sample was shown in Table 2. Differentially expressed tRFs and tiRNAs were screened based on their count values using the R package edgeR^13^. Hierarchical clustering and volcano plots were performed in R or perl environment.

### Overview of tRF and tiRNA expression in VCA

**Fig. 5:**
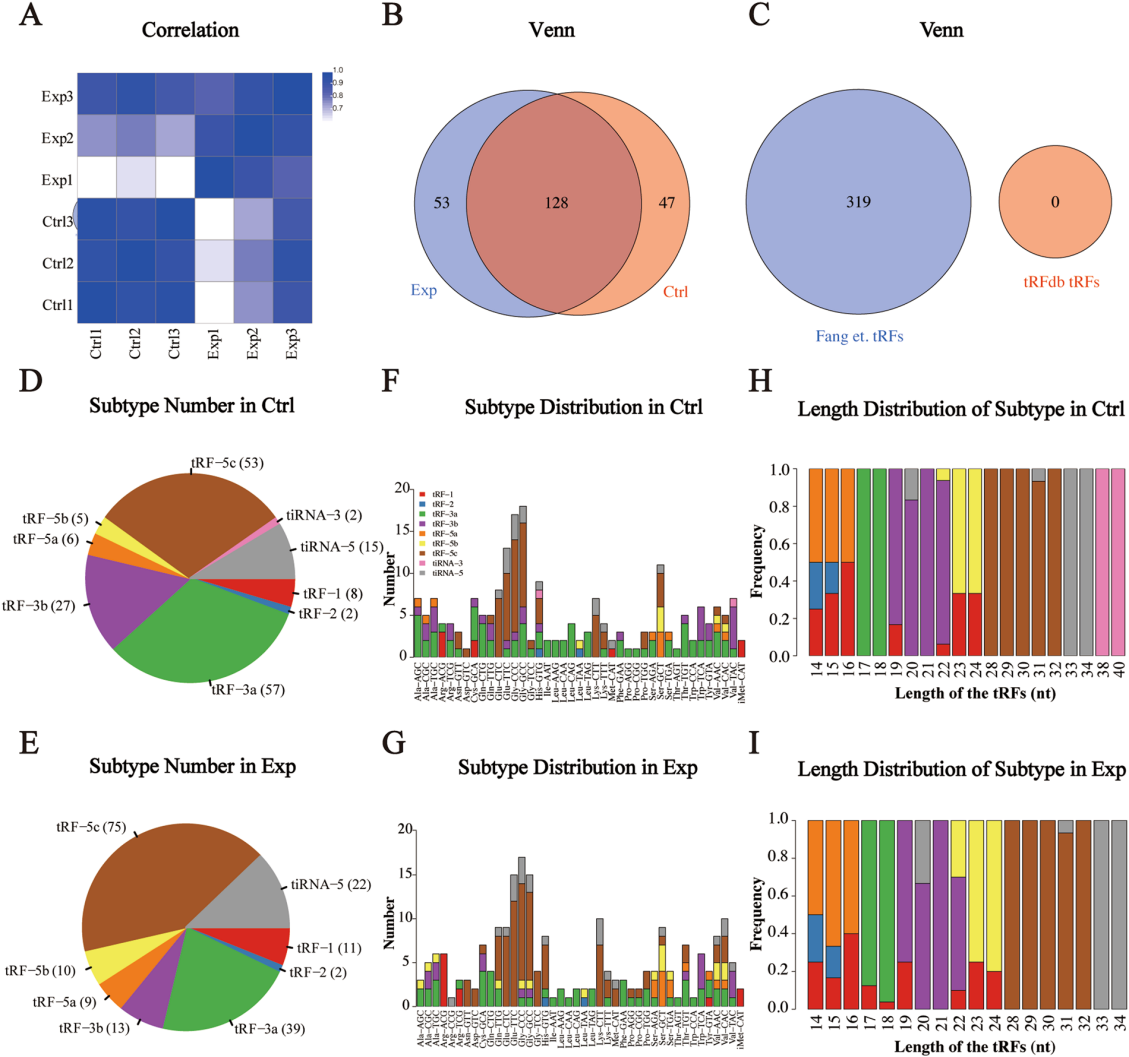
The analysis of tRF & tiRNA. (**A**) Heatmap of correlation coefficient from all samples. The color in the panel represents the correlation coefficient of the two samples. Blue represents the two samples with a high correlation coefficient, and the white represents the low similarity of the two samples. (**B**) Venn diagram based on number of commonly expressed and specifically expressed tRF & tiRNA. (**C**) Venn diagram based on number of tRFdb tRFs and Fang et. tRFs. tRFdb tRFs represent the tRFs from the tRFdb database and Fang et. tRFs represent the tRFs from our detected tRFs. (**D**,**E**) The distribution of tRF & tiRNA subtypes. (**F**,**G**) The number of subtype tRF & tiRNA against tRNA isodecoders. (**H**,**I**) The Frequency of Subtype against Length of the tRF & tiRNA.

The correlation coefficient between samples is a proxy of the reliability of the sample selections. The closer the coefficient is to 1, the more similar the correlation is between the two comparison samples. Thus, based on the expression level of each sample, we calculated the correlation coefficients between any two samples within all our samples (Fig. 5A). Conventional and specifically expressed tRFs and tiRNAs are depicted in the Venn diagram. 128 tRFs and tiRNAs were co-expressed in both groups, while 53 and 47 tRFs and tiRNAs were specifically expressed in the experimental and control groups, respectively (Fig. 5B). Figure 5C depicts the known tRFs and tiRNAs from the tRFdb (tRFdb tRFs) and the tRFs and tiRNAs detected in this project (Fang *et al*. tRFs)^14,15^. The figure indicates that the 319 tRFs and tiRNAs that we detected were unknown, which suggested that our experiments had identified many RNA fragments that are not included in the tRFdb database. We call them Fang *et al*. tRFs for the time being. The pie chart depicts the distribution of each tRF and tiRNA subtype. Notably, tRF-3a and tRF-5c form the majority in the two groups (Fig. 5D,E). tRNA isodecoders share the same anticodon but differ in their body sequences. The stacked bar graph illustrates the distribution of different tRNA subtypes (Fig. 5F,G). The frequencies of the tRFs and tiRNAs (CPM of the sample or average CPM of the group was not less than 20) could be calculated according to sequence length. The stacked bar graphs represent different tRF and tiRNA lengths. The height of the results bar represents combined tRF and tiRNA lengths (Fig. 5H,I).

### Differentially expressed tRFs and tiRNAs analysis

**Fig. 6:**
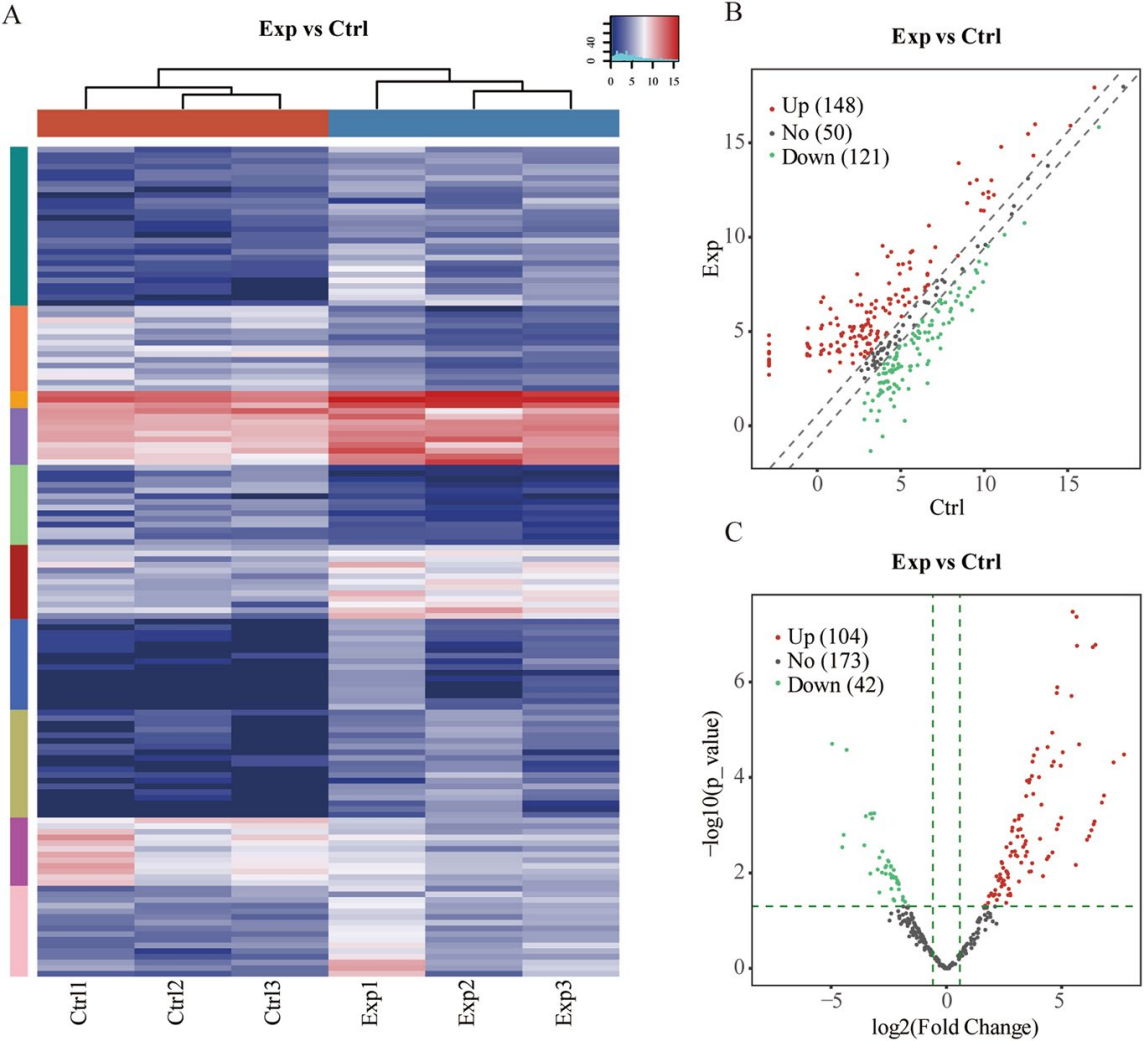
(**A**) The hierarchical clustering heatmap for tRF & tiRNA. (**B**) The scatter plot between two groups for tRF & tiRNA. (**C**) The volcano plot of tRF & tiRNA. (Red: up-regulated; Green: down-regulated).

Analyses of the differentially expressed tRFs and tiRNAs were performed using the R package edgeR (cutoff of 1.5). The p-values (cutoff of 0.05 performed only when replicates were available) were used for screening the differentially expressed tRFs and tiRNAs. Table 1 lists the differentially expressed tRFs and tiRNAs. We used visual methods to highlight the differences in expression between the tRFs and tiRNAs. Hierarchical clustering was applied to the group samples according to the expression levels of the samples. Figure 6A indicates that the control groups clustered together, while the experimental groups clustered together. It demonstrates that the two groups were largely different. Scatter plots could be used to evaluate changes in expression levels between the two sample groups (Fig. 6B). The red dots on the upper line represent up-regulated tRFs and tiRNAs, while the green dots on the lower line represent down-regulated tRFs and tiRNAs. The volcanic map could quickly identify differences between the two groups, and it indicated significant changes in up- and down-regulation (Fig. 6C).

**Table 1:** Differentially expressed tRFs and tiRNAs.

tRF_ID	Type	Length	Fold_Change	p_value	q_value	Regulation
tiRNA-1-34-Lys-CTT-1	tiRNA-5	34	13.76215506	3.45499E-05	0.000648318	Up
tiRNA-1-33-Gly-GCC-1	tiRNA-5	33	7.728215554	0.00079526	0.006040187	Up
tRF-1-32-Gly-GCC-2-M2	tRF-5c	32	7.22947955	0.001124944	0.007801241	Up
tiRNA-1-34-Glu-CTC-1	tiRNA-5	34	6.042244124	0.002893825	0.014441748	Up
tiRNA-1-33-Gly-CCC-1	tiRNA-5	33	5.212355372	0.005928161	0.023638543	Up
tRF-54-71-chrM.Cys-GCA	tRF-3a	18	0.113355132	0.000563443	0.005253418	Down
tRF-53-71-chrM.Cys-GCA	tRF-3b	19	0.187449861	0.007439451	0.027277986	Down
tRF-59-76-Val-AAC-1-M2	tRF-3a	18	0.187463176	0.007175748	0.026617019	Down
tRF-59-75-Gln-CTG-2-M2	tRF-3a	17	0.204078893	0.008680851	0.030430677	Down
tRF-58-74-Gly-CCC-1-M4	tRF-3a	17	0.208980617	0.01268498	0.037467673	Down
tRF-60-77-Ile-AAT-1-M2	tRF-3a	18	0.217010246	0.012456416	0.037467673	Down
tRF-60-76-Thr-TGT-2-M2	tRF-3a	17	0.225534264	0.016236385	0.043524427	Down
tRF-60-76-Phe-GAA-1-M3	tRF-3a	17	0.226571881	0.014434599	0.040480965	Down
tRF-59-75-Gln-CTG-1-M7	tRF-3a	17	0.229756322	0.016022911	0.043524427	Down
tRF-60-76-Val-AAC-1-M4	tRF-3a	17	0.233552323	0.015982978	0.043524427	Down
tRF-58-75-Gln-CTG-2-M2	tRF-3a	18	0.236884088	0.017221421	0.045780277	Down

**Table 2:** Sequencing reads.

Sample	TotalRead	Trimmed	Mat-tRNA	Mat-tRNA(%)	Pre-tRNA	Pre-tRNA(%)
Ctrl 1	8344966	7420099	536416	7.23	13588	0.18
Ctrl 2	9784210	8275188	700344	8.46	40283	0.49
Ctrl 3	8020391	6991904	302818	4.33	14172	0.20
Exp 1	7292491	4804767	847441	17.64	29053	0.60
Exp 2	8018823	6808582	1891337	27.78	35932	0.53
Exp 3	8242986	6235736	1835763	29.44	30464	0.49

### Bioinformatic analysis

**Fig. 7:**
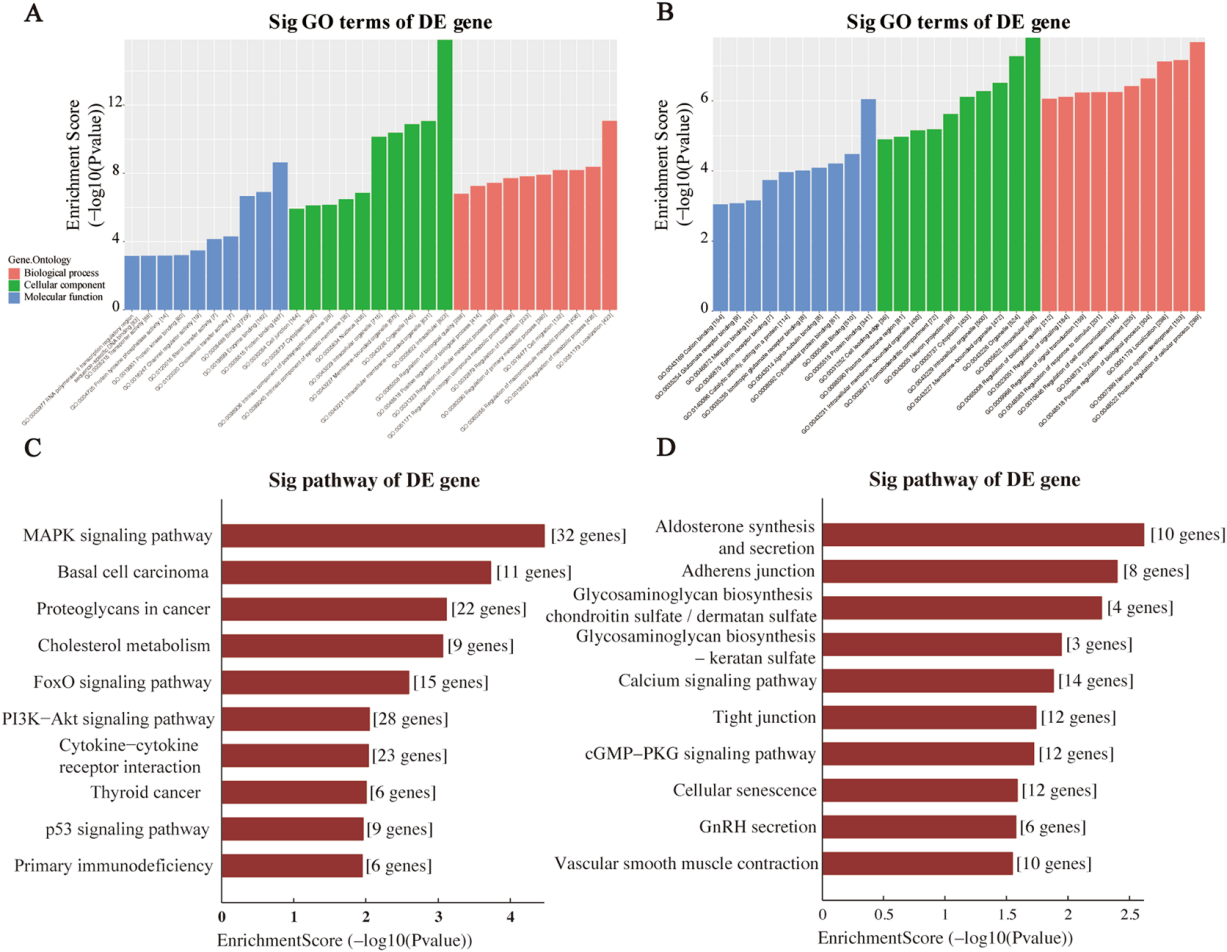
Bioinformatic Prediction. GO (**A**) and Pathway (**C**) analysis for up-regulated tRF & tiRNA. GO (**B**) and Pathway (**D**) analysis for down-regulated tRF & tiRNA.

The selection of tRF & tiRNA criteria included a higher FC, lower p-value and higher CMP. After a unified summary of the original data, 16 differentially expressed tRFs & tiRNAs were screened, among which 5 tRFs & tiRNAs were significantly up-regulated and 11 tRFs & tiRNAs were significantly down-regulated. The following two algorithms were integrated. The first was Miranda, an old dynamic programming algorithm based on RNA secondary structures and free energy^16^. The second was TargetScan, which can identify biologically significant site sequence characteristics and build relatively conservative scoring models according to the fit of the mRNA and tRF expression profile data^17,18^. However, it can only search for perfect matching sites of nucleotides 2–7 in length such as M8, 7mer-m8, and 7mer-a1. By combining the two algorithms and integrating their capabilities, the target genes were obtained for Gene Ontology (GO) and Kyoto Encyclopedia of Genes and Genomes (KEGG) analysis. The GO project provides a controlled vocabulary to describe gene and gene product attributes in any organism (http://www.geneontology.org). The ontology covers three domains: Biological Process, Cellular Component and Molecular Function. Fisher’s exact test in Bioconductor’s top GO is used to find if there is more overlap between the DE list and the GO annotation list than would be expected by chance. The p-value produced by top GO denotes the significance of GO terms enrichment in the DE genes. The lower the p-value, the more significant the GO Term. Pathway analysis is a functional analysis mapping genes to KEGG pathways. The p-value (EASE-score, Fisher-Pvalue or Hypergeometric-Pvalue) denotes the significance of the Pathway correlated to the conditions. Lower the p-value, more significant is the Pathway. The top 10 significantly enriched GO annotations for the predicted targets of up-regulated tsRNAs are listed in Fig. 7A. Figure 7A,B indicates that both groups were mainly enriched in Intracellular and Protein Binding. The MAPK pathway has the highest enrichment score and the most genes (Fig. 7C). Down-regulated tRFs and tiRNAs were mainly enriched in Calcium signaling pathway and cGMP-PKG signaling pathway (Fig. 7D).

### Reverse transcription quantitative polymerase chain reaction (RT-qPCR)

The rtStar™ First-Strand cDNA Synthesis Kit (3′ and 5′ adaptor) (Cat# AS-FS-003, Arraystar) was used to synthesize cDNA. The QuantStudio™ 5 Real-time PCR System (Applied Biosystems) was used for the RT-qPCR. Relative tsRNA levels and CACNA1D gene level were normalized according to U6 snRNA, and the mean and standard error were calculated. The primer sequences that we used are listed in Table 3.

**Table 3:** Sequences of primers for RT-qPCR.

Gene_ID	Primer sequences	Ta Opt (°C)	Product size (bp)
U6	F:5′GCTTCGGCAGCACATATACTAAAAT3′	60	89
	R:5′CGCTTCACGAATTTGCGTGTCAT3′		
tiRNA-1-33-Gly-GCC-1	F:5′ GATCGCATTGGTGGTTCAGTG 3′	60	48
	R:5′ TCTTCCGATCTCAGGCGAGAA 3′		
tiRNA-1-34-Glu-CTC-1	F:5′ ACAGTCCGACGATCTCCCTGG 3′	60	51
	R:5′ TCTGAGCGCCGAATCCTAACC 3′		
tRF-1-32-Gly-GCC-2-M2	F:5′ GATCGCATGGGTGGTTCAGT 3′	60	48
	R:5′ CTCTTCCGATCTAGGCGAGAAT 3′		
tRF-59-75-Gln-CTG-2-M2	F:5′ CTACAGTCCGACGATCTCTCG 3′	60	45
	R:5′ CTCTTCCGATCTTGGAGGTTC 3′		
tRF-59-76-Val-AAC-1-M2	F:5′ CTACAGTCCGACGATCAACCG 3′	60	46
	R:5′ CTCTTCCGATCTTGGTGTTTCC 3′		
CACNA1D	F:5′ CATCACCACCTTGTAGCCCA 3′	60	124
	R:5′ TGAGCATTTTCCTCCCAACTA 3′		

**Fig. 8:**
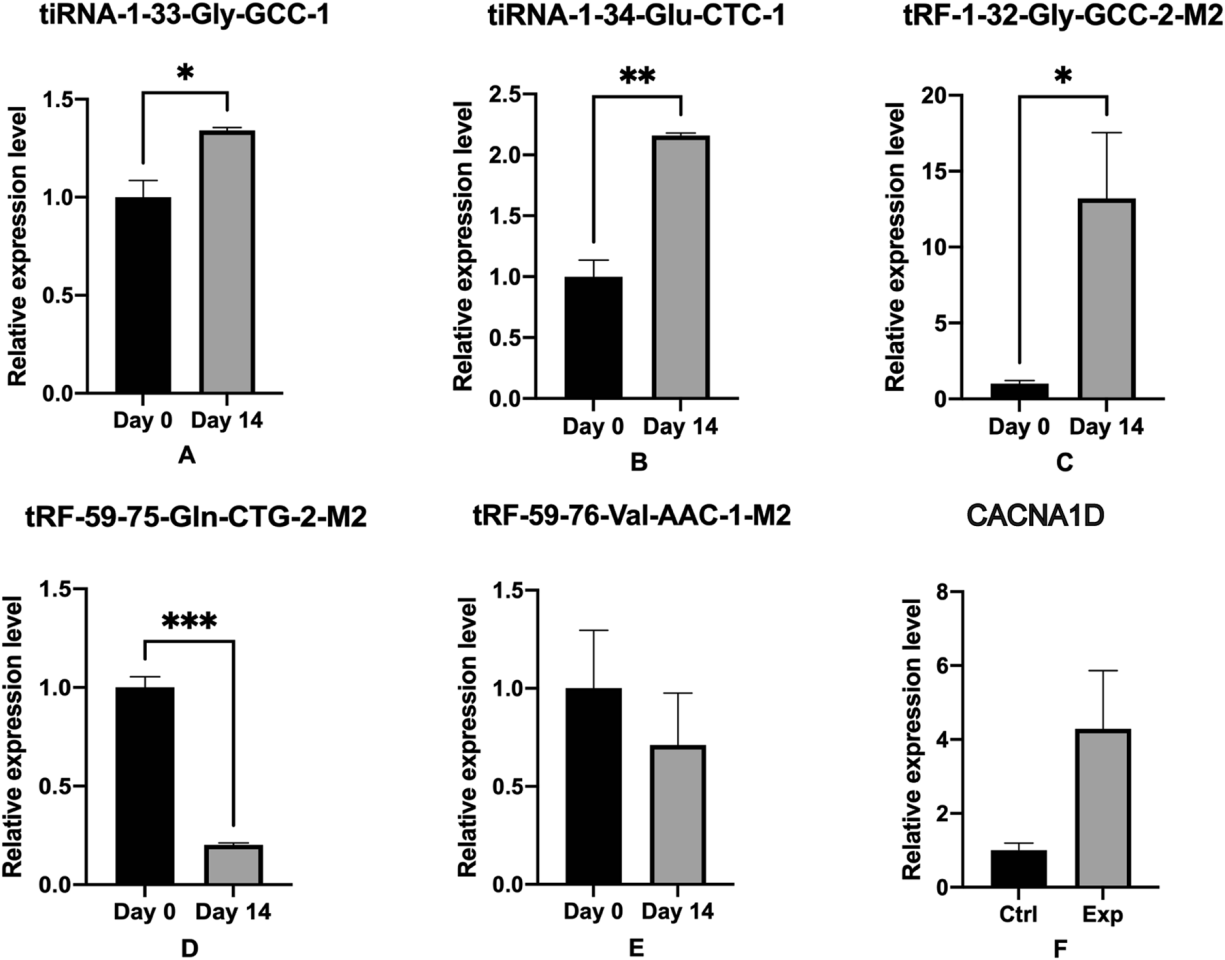
RT-qPCR verification. Compared with the control gourp (day 0), (**A**–**C**) tiRNA-1:33-Gly-GCC-1, tiRNA-1-34-Glu-CTC-1 and tRF-1-32-Gly-GCC-2-M2 were up-regulated; (**D**,**E**) tRF-59-75-Gln-CTG-2-M2 and tRF-59:76-Val-AAC-1-M2 were down-regulated. (**F**) The relative expression level of CACNA1D gene. The data were normalized using the mean ± standard error of the mean (SEM). *P ≤ 0.05, **P ≤ 0.01, ***P ≤ 0.001.

Among the five up-regulated tRFs and tiRNAs, we selected three tsRNAs that had the most target genes in the MAPK pathway. Similarly, we selected two tsRNAs with the most target genes in Calcium pathway. The five tsRNAs and CACNA1D gene were verified using RT-qPCR, and the results were depicted in Fig. 8. Their relative expression levels were statistically analysed using t-tests. P ≤ 0.05 was considered statistically significant.

## Data Records

The Raw_Data files and Aligment datasets presented in this study can be found in online repositories. The names of the repository/repositories and accession number(s) can be found below: Gene Expression Omnibus (GEO) database under accession number GSE190008^19^. All submitted data can be found in the GSE190008 including raw data and original data.

## Technical Validation

### RNA-seq data quality assessment

Raw data files in FASTQC format were generated from the Illumina sequencer. To examine the sequencing quality, the quality score plot of each sample was plotted (Fig. 4). Quality score Q is logarithmically related to the base calling error probability (P):

$${\boldsymbol{Q}}=-{\bf{10}}{{\boldsymbol{log }}}_{{\bf{10}}}({\boldsymbol{P}})$$

## Data Availability

Public domain software tools were used in tRF & tiRNA-seq quality verification and standardized naming. These software tools are listed below: Sequencing quality are examined by FastQC: https://www.bioinformatics.babraham.ac.uk/projects/fastqc/. The standardized tRF & tiRNA ID for tRNA derived fragments consists of four parts: http://trna.ucsc.edu/tDRnamer/.
